# Zebrafish nephrosin helps host defence against *Escherichia coli* infection

**DOI:** 10.1098/rsob.170040

**Published:** 2017-08-23

**Authors:** Qianqian Di, Qing Lin, Zhibin Huang, Yali Chi, Xiaohui Chen, Wenqing Zhang, Yiyue Zhang

**Affiliations:** 1 Key Laboratory of Zebrafish Modeling and Drug Screening for Human Diseases of Guangdong Higher Education Institutes, Department of Developmental Biology, School of Basic Medical Sciences, Southern Medical University, Guangzhou 510515, People's Republic of China; 2 Laboratory of Developmental Biology and Regenerative Medicine, School of Medicine, South China University of Technology, Guangzhou 510006, People's Republic of China

**Keywords:** neutrophil, nephrosin, infection, zebrafish

## Abstract

Neutrophils play important roles in innate immunity and are mainly dependent on various enzyme-containing granules to kill engulfed microorganisms. Zebrafish *nephrosin* (*npsn*) is specifically expressed in neutrophils; however, its function is largely unknown. Here, we generated an *npsn* mutant (*npsn^smu5^*) via CRISPR/Cas9 to investigate the *in vivo* function of Npsn. The overall development and number of neutrophils remained unchanged in *npsn*-deficient mutants, whereas neutrophil antibacterial function was defective. Upon infection with *Escherichia coli*, the *npsn^smu5^* mutants exhibited a lower survival rate and more severe bacterial burden, as well as augmented inflammatory response to challenge with infection when compared with wild-type embryos, whereas *npsn*-overexpressing zebrafish exhibited enhanced host defence against *E. coli* infection. These findings demonstrated that zebrafish Npsn promotes host defence against bacterial infection. Furthermore, our findings suggested that *npsn*-deficient and -overexpressing zebrafish might serve as effective models of *in vivo* innate immunity.

## 1. Introduction

Neutrophils constitute the most abundant circulating leukocytes and play important roles in the innate immune system, with essential functions related to host defence against invading pathogens [1]. Upon host infection, neutrophils are typically the first responders recruited from haematopoietic tissue and travel through the vasculature to infected sites [2]. Neutrophils primarily eliminate pathogens in two ways: (i) secretion of cytokines and chemokines, such as interleukin (IL)-1b [3], IL-18 [4] and IL-37 [5], to recruit and activate additional phagocytes to the infection site; and (ii) phagocytosing microbes, primarily dependent on their granules [6]. Neutrophils contain the following four types of granules: azurophil (also known as primary) granules, specific (also known as secondary) granules, gelatinase (also known as tertiary) granules and secretory granules [7]. These granules destroy pathogens by either activating membrane-bound NADPH oxidase to generate reactive oxygen species [8,9] or through proteolysis to destroy the integrity of bacterial membranes or cytoderms [10,11]. However, the function of these granules has mainly been studied *in vitro*, leaving many of their *in vivo* functions unknown.

Nephrosin (Npsn) was first discovered in the lymphohaematopoietic tissues of *Cyprinus carpio* and is a zinc metalloendopeptidase belonging to the astacin family [12], which exhibit diverse biological functions including protein digestion, dorsal/ventral determination and morphogenesis [13]. Carp Npsn exhibits greater than 50% sequence identity to medaka-hatching enzymes, although there was no *npsn* expression observed in carp hatching liquid, indicating that Npsn is not a hatching-enzyme analogue [12]. Another study found that *npsn* was specifically expressed at zebrafish haematopoietic sites [14], and most *npsn*-positive cells express granulocytic markers [14], suggesting that zebrafish Npsn might be a granzyme in granulocytes. However, the function of Npsn in granulocytes remains unknown.

The zebrafish (*Danio rerio)* has emerged as a powerful vertebrate model for the study of infectious disease. Despite its traditional advantages, including high fecundity, external development and convenient tools capable of manipulating gene expression, zebrafish offer many unique advantages for studying neutrophils and pathogens. First, the vertebrate innate immune system is highly conserved between zebrafish and mammals, including their containing macrophages [15], neutrophils [16,17] and complements [18]. Additionally, their establishment of a functional adaptive immune system is delayed until approximately 3 weeks post-fertilization [19]. This distinction in immune development makes zebrafish embryos and larvae ideally suited to study host innate immune response to bacterial pathogens [20,21], as well as functions and behaviours of neutrophils in microbe infection [22,23]. Second, the process of granulocytopoiesis, involving origination from haematopoietic stem cells and development into myeloblasts and mature granulocytes, is conserved between mammals and zebrafish [24,25]. Additionally, zebrafish models allow the unique ability to study host–pathogen interactions in real time due to the transparency of zebrafish embryos and the wide range of available fluorescence-analysis tools [26]. Therefore, the zebrafish represents an ideal system for studying the function of Npsn in inflammatory response.

In this study, we used the CRISPR/Cas9 system to obtain an *npsn*-deficient mutant (*npsn^smu5^*) in order to investigate the *in vivo* function of Npsn. The mutant showed no altered neutrophil number, but exhibited deficient antibacterial function. Upon infection with *Escherichia coli*, the *npsn^smu5^* mutants exhibited a lower survival rate and more severe bacterial burden, as well as increased inflammatory response to challenge with infection, when compared with wild-type (WT) embryos. Additionally, we observed that *npsn* overexpression enhanced host defence against *E. coli* infection. Our findings suggested that Npsn is crucial for host defence against bacterial infection, and that *npsn*-deficient and overexpressing zebrafish might serve as effective models of *in vivo* innate immunity.

## 2. Results

### 2.1. Zebrafish *npsn* is expressed in neutrophils

**Figure 1.**
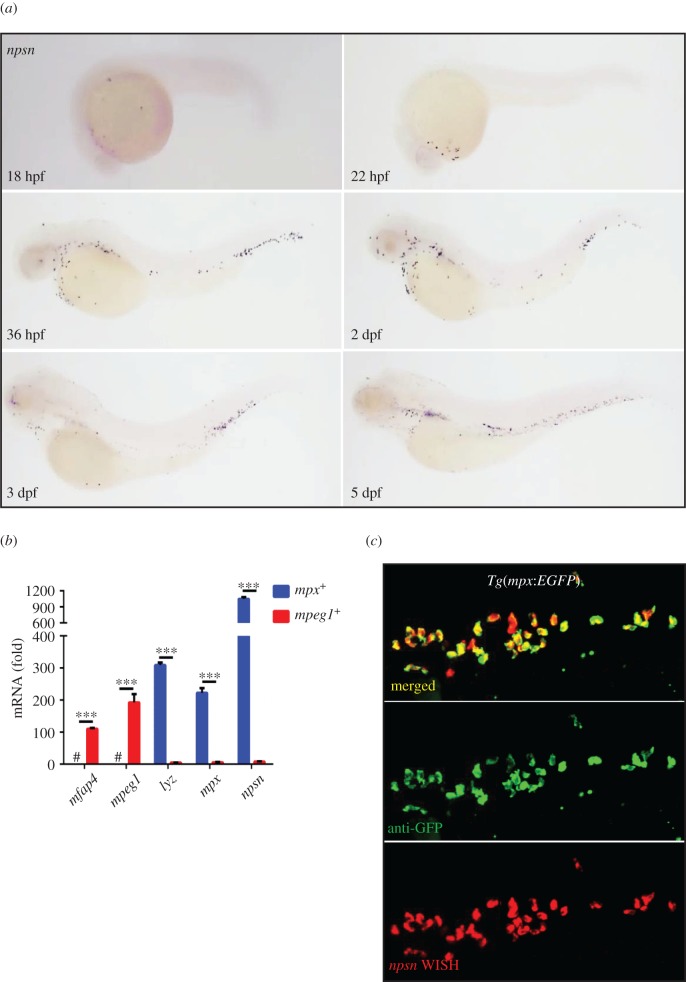
Zebrafish *npsn* is predominantly expressed in neutrophils. (*a*) The expression pattern of *npsn* during zebrafish embryogenesis was initially observed at the rostral blood island at 18 hpf, followed by widespread expression across the site of haematopoiesis in zebrafish. (*b*) Higher expression of *npsn* in neutrophils when compared with macrophages. Fluorescent cells were sorted from *Tg(mpx:EGFP)* and *Tg(mpeg1:EGFP)*, and qRT-PCR was performed to detect the *npsn* expression, which was highly expressed in neutrophils relative to levels observed in macrophages following adjustment for GFP^−^ cells. Macrophage markers (*mfap4* and *mpeg1*) and neutrophil markers (*lyz* and *mpx*) were used as indicators to test the purity of the sorted macrophages and neutrophils; ***p < 0.001. #, undetected. (Mean ± s.e.m., *n* ≥ 200 per experiment, triplicated). (*c*) Co-staining for *npsn* mRNA in *mpx^+^* cells at the posterior blood island (PBI) in *Tg(mpx:EGFP)* embryos. Double staining for anti-GFP and *npsn* WISH was performed in *Tg(mpx:EGFP)* cells at 3 dpf. Most *mpx^+^* neutrophils also expressed *npsn* mRNA in the PBI.

To determine the role of Npsn in embryogenesis, we examined the temporal and spatial expression of *npsn* by whole-mount *in situ* hybridization (WISH) analysis. At 18 h post-fertilization (hpf), we observed that *npsn* was initially expressed in the rostral blood island, the location of myelopoiesis in primitive haematopoiesis, then spread widely across the site of haematopoiesis (figure 1*a*), which was consistent with findings from a previous study [14]. To identify *npsn* expression in myeloid cells, fluorescent cells sorted from neutrophil-specific *Tg(mpx:EGFP)* and macrophage-specific *Tg(mpeg1:EGFP)* zebrafish lines were used to detect the relative expression of *npsn* in neutrophils and macrophages, respectively. Our results showed that *npsn* was highly expressed in neutrophils and relatively much lower in macrophages after adjustment for GFP^−^ cells (figure 1*b*). Co-staining with anti-GFP and *npsn* WISH analysis of *Tg(mpx:EGFP)* confirmed that most *npsn^+^* cells could be co-stained with *mpx^+^* neutrophils (figure 1*c*), demonstrating neutrophil-specific expression.

Based on this expression pattern, we cloned the *npsn* promoter to drive GFP expression in order to generate a zebrafish reporter allowing visualization of *npsn*^+^ cells. We isolated a 2 kb DNA fragment upstream of the *npsn*-translation start site in the promoter region to drive GFP expression in the Tol2 vector (referred to as *pTol2-npsn-EGFP*) (electronic supplementary material, figure S1*a*). The *pTol2-npsn-EGFP* construct was injected into one-cell-stage WT embryos, followed by screening for founders capable of producing offspring exhibiting GFP expression (referred to as *Tg(npsn:EGFP)smu7*) at developing haematopoietic sites (electronic supplementary material, figure S1*b*). To determine whether GFP^+^ cells could report *npsn* expression, we performed double fluorescent staining for GFP and *npsn* WISH, with results showing that the majority of the fluorescent signals were co-located (electronic supplementary material, figure S1*c*). Inter-crossing the *Tg(npsn:EGFP)smu7* line with the *Tg(lyz:DsRed)* line indicated that most of the *npsn^+^* cells overlapped with *lyz^+^* cells in the haematopoietic regions, including the thymus (electronic supplementary material, figure S1*d*, 1*d*′), aorta–gonad–mesonephros (electronic supplementary material, figure S1*e*,1*e*′) and posterior blood island (PBI) (electronic supplementary material, figure S1*f*,1*f*′). To compare the specificity of *Tg(npsn:EGFP)smu7* and *Tg(mpx:EGFP)*, we sorted the GFP^+^ cells, respectively, by flow cytometry, with quantitative reverse transcription polymerase chain reaction (qRT-PCR) results showing that the expression levels of neutrophil markers (*mpx*, *lyz* and *npsn*) were significantly higher in *npsn^+^* cells, whereas levels of macrophage markers (*mfap4* and *mpeg1*) were lower (electronic supplementary material, figure S1*g*), suggesting that the *npsn* promoter drove expression specifically in neutrophils. Taken together, these findings suggested that *npsn* was predominantly expressed in neutrophils rather than in macrophages.

### 2.2. Generation and identification of *npsn*-deficient zebrafish

**Figure 2.**
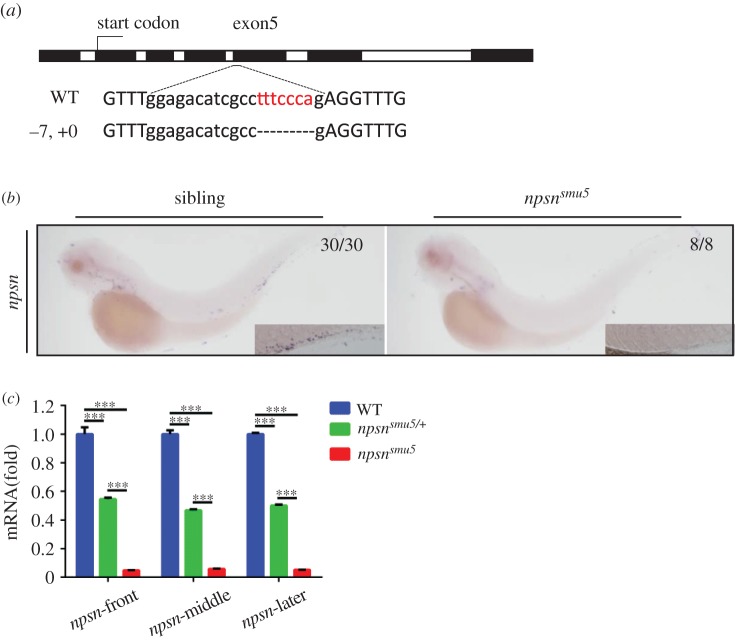
Generation and identification of *nps-*deficient zebrafish. (*a*) The CRISPR/Cas9 system was used to generate *npsn*-knockout zebrafish. The Cas9 target was chosen as exon5 of *npsn*. Mutants were selected using T7E1 enzyme digestion and confirmed using Sanger sequencing. In F1 founders, a mutant line (−7, +0) containing a frameshift mutation was obtained and retained for follow-up studies. (*b* and *c*) Degradation of *npsn* mRNA in *npsn^smu5^* mutant embryos. (b) WISH results for *npsn* mRNA indicated significant decreases in *npsn^smu5^* mutant embryos at 3 dpf. (*c*) qRT-PCR results indicating that *npsn* mRNA expression was downregulated to 5% in *npsn^smu5^* mutant embryos. (Mean ± s.e.m., *n* = 20 per experiment, triplicated). Statistical significance was determined by one-way ANOVA. ***p < 0.001.

To characterize the *in vivo* function of Npsn in zebrafish neutrophils, we generated *npsn*-knockout lines utilizing the CRISPR/Cas9 system. We obtained two frameshift mutations, with one (−7, +0) in exon5-Cas9 (referred to as *npsn^smu5^*) (figure 2*a*) and the other (−0, +1) in exon6-Cas9 (referred as *npsn^smu6^*) (electronic supplementary material, figure S2*a*). We then performed WISH to detect the expression of *npsn* mRNA. Interestingly, *npsn* expression was significantly decreased in *npsn^smu5^* and *npsn^smu6^* embryos at various stages of development (24 hpf, 36 hpf, 2 days post-fertilization (2 dpf) and 3 dpf) (figure 2*b* and electronic supplementary material, figure S2*b*,*c*). Levels of *npsn* mRNA exhibited similar downregulation to 5% in homozygous *npsn^smu5^* embryos, with a 50% decrease in the heterozygote (figure 2*c*), suggesting that *npsn* mRNA may undergo full-scale degradation in *npsn^smu5^* embryos, with reductions in *npsn* mRNA possibly due to nonsense-mediated decay and consistent with a well-identified mechanism for elimination of mRNA containing premature stop codons [27]. The *npsn^smu5^* and *npsn^smu6^* homozygotes were able to survive normally to adulthood, with further observation and breeding indicating that *npsn*-deficient zebrafish were morphologically indistinguishable from their heterozygous and WT siblings and capable of normal reproduction.

### 2.3. *npsn* deficiency does not affect neutrophil number in zebrafish

**Figure 3.**
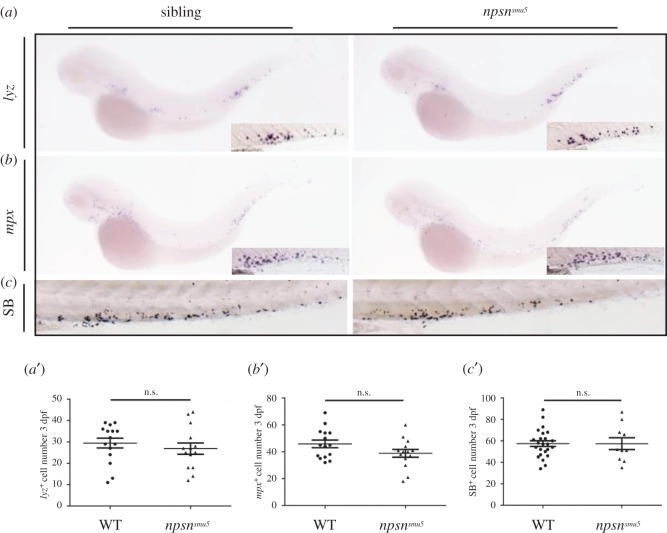
*npsn* deficiency does not affect neutrophil number in zebrafish. (*a* and *a*′) WISH analysis of *lyz* expression and quantification of *lyz^+^* cells in the PBI at 3 dpf (29.5 ± 2.3 versus 26.9 ± 2.7) in WT sibling and *npsn^smu5^* mutant groups. (Mean ± s.e.m., *n* = 15 in each group, triplicated). Boxes in the lower right corner outline the magnified PBI regions. (*b* and *b*′) WISH analysis of *mpx* expression and quantification of *mpx^+^* cells in the PBI at 3 dpf (45.9 ± 2.9 versus 38.9 ± 2.9) in WT sibling and *npsn^smu5^* mutant groups. (Mean ± s.e.m., *n* = 15 in each group, triplicated). Boxes in the lower right corner outline the magnified PBI regions. (*c* and *c*′) Sudan Black staining (SB) and quantification of SB^+^ cells in the PBI at 3 dpf (57.5 ± 2.7 versus 57.4 ± 5.4) in WT sibling and *npsn^smu5^* mutant groups). (Mean ± s.e.m., *n* ≥ 10 in each group, triplicated). Statistical significance was determined by unpaired *t*-test. n.s., not significant.

To determine whether loss of Npsn causes neutrophil defects, we examined the expression of neutrophil-specific genes. WISH results for *mpx* and *lyz* showed no difference in the expression of neutrophil markers between WT siblings and *npsn^smu5^* embryos (figure 3*a*,*a*′,*b*,*b*′). According to Sudan Black B staining, we also found no SB^+^ cell changes in the mutants (figure 3*c*,*c*′), suggesting unaltered neutrophil number in *npsn^smu5^* mutants. We then examined neutrophil granules by differential-interference contrast microscopy and found that granules were intact in *npsn^smu5^* embryos and WT siblings (data not shown), suggesting no visible developmental defects of neutrophils in *npsn^smu5^* mutants. These observations indicated that *npsn* deficiency did not cause developmental defects or affect neutrophil distribution.

### 2.4. *npsn* deficiency affects host defence against *E. coli* infection

**Figure 4.**
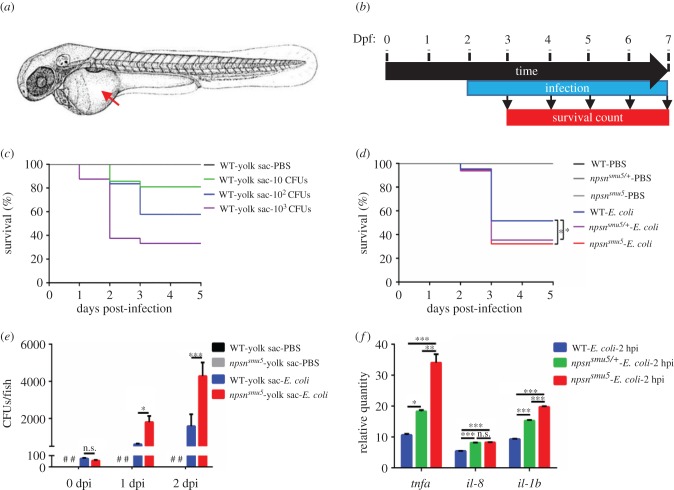
*npsn* deficiency affects host defence against *E. coli* infection. (*a*) The infection site of the zebrafish yolk sac (the red arrow). (*b*) Scheme showing the experimental procedure used for survival assays. The *npsn^smu5^* mutants and WT controls were infected with *E. coli* at 2 dpf via the yolk sac, and the number of surviving larvae was counted daily over the next 5 days. At least three independent experiments were performed using greater than 60 embryos per group. (*c*) Survival curves of WT embryos challenged with different doses of *E. coli*. Mortality increased in a dose-dependent manner. (*d*) Survival curves of *npsn^smu5^* and WT embryos injected with 100 CFUs of *E. coli* (WT (*n* = 57); *npsn^smu5^* (*n* = 55) in total). Statistical significance was determined by the log-rank test. *p < 0.05. (*e*) Bacterial burden of embryos injected with *E. coli*. Significantly more bacterial cells were observed in *npsn^smu5^* mutant embryos when compared with those observed in WT controls at 1 and 2 dpi. Data were combined from three individual experiments (*n* = 50 per group), and statistical significance was determined using the two-way ANOVA with Bonferroni's multiple comparisons adjustment. *p < 0.05. ***p < 0.001. n.s., not significant. #, undetected. (*f*) Alteration of the inflammatory response in *npsn^smu5^* embryos at 2 hpi. The relative quantity of inflammatory factors *il-1b*, *il-8* and *tnfα* was examined by qRT-PCR, and expression levels were adjusted for trauma (PBS-solution injection). (Mean ± s.e.m., *n* = 30 in each group, triplicated). Statistical significance was determined using the one-way ANOVA with Bonferroni's multiple comparisons adjustment. *p < 0.05. **p < 0.01. ***p < 0.001. n.s., not significant.

Neutrophils are indispensable for host defence against intruding microorganisms. To determine whether *npsn* deficiency affects zebrafish defence against bacterial infection, we infected WT and *npsn^smu5^* embryos in the yolk sac with *E. coli* as described previously [28] (figure 4*a*,*b*). First, the survival rate of infected embryos was determined to evaluate the appropriate infective dose, with results indicating that as few as 10 colony forming units (CFUs) of bacteria could be lethal to WT embryos in a dose-dependent manner (figure 4*c*), whereas 100 CFUs resulted in a survival rate of between approximately 40% and approximately 60%, resulting in its subsequent selection as a proper dosage for follow-up studies. We then detected whether *npsn* level was regulated by *E. coli* infection, and found that the expression of *npsn* was unaffected in neutrophils of bacteria-injected embryos compared with phosphate-buffered saline (PBS)-injected controls (electronic supplementary material, figure S3). All *npsn^smu5^* homozygous embryos, *npsn^smu5/+^* heterozygous embryos and WT embryos survived normally at 1 day post-infection (dpi), with death occurring at either 2 or 3 dpi. However, both the *npsn^smu5^* and *npsn^smu5/+^* embryos exhibited a significantly lower survival rate relative to their WT embryos at 3 dpi (figure 4*d*). These data indicated that *npsn* deficiency weakened host resistance to *E. coli* infection.

We then measured kinetic curves associated with *in vivo* bacterial growth of infected embryos. Our results showed that by 2 dpi, *E. coli* proliferated in both WT and *npsn^smu5^* embryos, and that there was a significantly higher bacterial burden in *npsn^smu5^* mutant embryos at 1 and 2 dpi (figure 4*e*). These results revealed that *E. coli* proliferated faster in mutant embryos, implying that *npsn* deficiency affected host defence against *E. coli* infection. Collectively, these findings revealed that *npsn* deficiency impaired neutrophil-specific antibacterial response in zebrafish embryos.

To further investigate infection-induced alterations in inflammation, we detected the expression of inflammatory factors, such as tumour necrosis factor α (*tnfα*) [29], *il-8* [30] and *il-1b* [31], during the early stage of infection, which could activate and induce neutrophil translocation to the infection site. Expression analysis by qRT-PCR at 2 hpi showed elevated expression of all genes to a more significant level in infected *npsn^smu5^* embryos and *npsn^smu5/+^* embryos than that in infected WT embryos and PBS-injected controls (figure 4*f*), suggesting a more severe inflammatory response in *npsn^smu5^* embryos. These findings clarified the changes in inflammatory response in *npsn^smu5^* mutants following *E. coli* infection.

**Figure 5.**
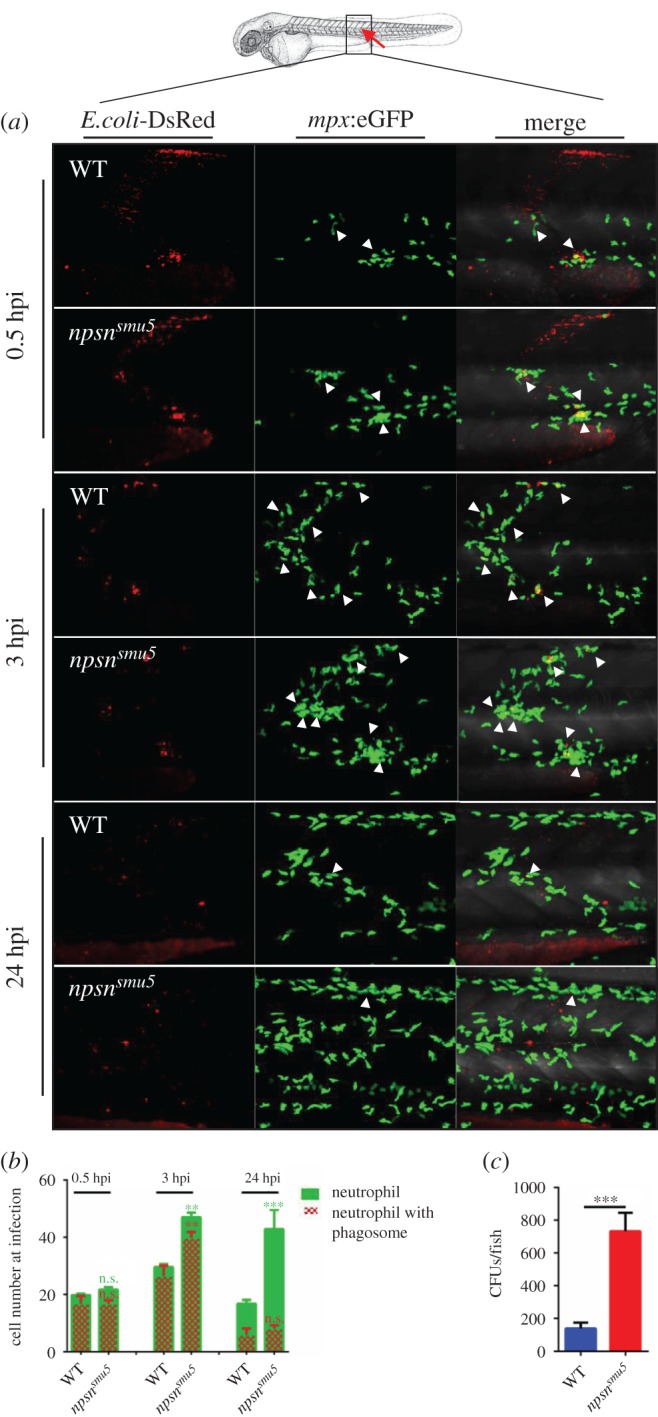
*npsn* deficiency affects neutrophil recruitment to bacteria. (*a*) The infection site of the zebrafish muscle (the red arrow). DsRed^+^ *E. coli* were injected subcutaneously over one somite into WT and *npsn^smu5^* mutant embryos with *Tg(mpx:eGFP)* background, and images were captured at 0.5, 3 and 24 hpi. All images are maximum-intensity projection at an interval of 2 µm. The white triangle indicated the neutrophil with phagosomes. (*b*) Quantification of recruited *mpx:eGFP^+^* neutrophil numbers (green bar) and phagocytosing neutrophil numbers (red net bar) in the infection site at each time point in bacterial injected WT and *npsn^smu5^* mutants. Average numbers with means in WT and *npsn^smu5^* mutant groups at 0.5, 3, 24 hpi (green bar: 19.7 ± 2.6 versus 21.7 ± 0.8; 28.6 ± 4.1 versus 47.0 ± 2.3; 17.5 ± 4.8 versus 42.8 ± 7.5); (red net bar: 16.9 ± 2.6 versus 16.83 ± 1.2; 26.4 ± 3.6 versus 39.8 ± 2.1; 5.9 ± 2.2 versus 8.2 ± 1.1). (Mean±s.e.m., *n* ≥ 6 in each group, triplicated). Statistical significance was determined using the two-way ANOVA with Bonferroni's multiple comparisons adjustment. **p < 0.01. ***p < 0.001. n.s., not significant. (*c*) Bacterial burden of embryos at 24 hpi in *npsn^smu5^* mutant embryos and WT controls. (Mean±s.e.m., *n* = 50 in each group, triplicated). Statistical significance was determined using the unpaired *t*-test. ***p < 0.001.

To further investigate whether neutrophil recruitment was affected in the *npsn^smu5^* embryos, we performed muscle infection in WT and *npsn^smu5^* embryos as previously described [22]. In WT embryos, 10–20 neutrophils first arrived at the infection site at 0.5 hpi, and the number increased to 25–35 at 3 hpi, which is consistent with previous observations [22]. As bacteria clearance completed, only 15–25 neutrophils were still at the infected site at 24 hpi (figure 5*a*,*b*). In the early stage of infection (0.5 hpi), *npsn^smu5^* embryos showed similar recruitment of neutrophils to WT embryos. However, recruited neutrophils in *npsn^smu5^* embryos were much more than in WT at 3 hpi, and were still accumulated without relief by 24 hpi (figure 5*a*,*b*). The number of neutrophils with phagosomes also increased in the *npsn^smu5^* embryos at 3 hpi (figure 5*b*). These results showed that *npsn^smu5^* embryos required more neutrophils to be recruited to form more phagosomes in clearance of bacteria, confirming that *npsn-*deficient embryos had severe inflammatory response. We further measured bacterial cells in infected embryos through muscle infection, and the results showed that there was a significantly higher bacterial burden in *npsn^smu5^* mutant embryos at 24 hpi (figure 5*c*), implying that *npsn* deficiency affected host defence against *E. coli* infection.

### 2.5. *npsn* overexpression enhances host immune response against *E. coli* infection

**Figure 6.**
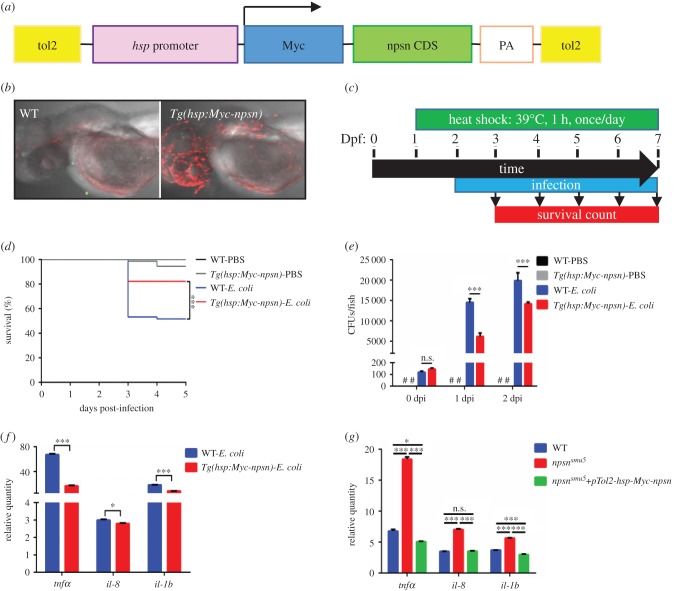
*npsn* overexpression enhances host response against *E. coli* infection. (*a*) Construction of the *Tg(hsp:Myc-npsn)* transgenic plasmid. *npsn*-CDS (green bar) was inserted behind the *hsp* promoter and a Myc tag. (*b*) Anti-Myc staining of the *Tg(hsp:Myc-npsn)* and WT embryos. (*c*) Scheme showing the experimental procedure used for the survival assays. *Tg(hsp:Myc-npsn)* and WT embryos were heat shocked at 39°C for 1 h at 1 day prior to infection. Embryos were infected with *E. coli* at 2 dpf via the yolk sac and heated to 39°C for 1 h daily, and the number of surviving larvae was counted daily over the next 5 days. (*d*) Survival curves for *Tg(hsp:Myc-npsn)* and WT embryos following injection with 100 CFUs of *E. coli* (WT (*n* = 60); *Tg(hsp:Myc-npsn)* (*n* = 60)). Statistical significance was determined using the log-rank test. ***p < 0.001. (*e*) Bacterial burden of embryos injected with *E. coli*. Less bacterial cells in *Tg(hsp:Myc-npsn)* at 1 and 2 dpi than in WT. Data were combined from three individual experiments (*n* = 50 per group), and statistical significance was determined using the two-way ANOVA with Bonferroni's multiple comparisons adjustment. ***p < 0.001. n.s., not significant. #, undetected. (*f*) Alteration of the inflammatory response in *Tg(hsp:Myc-npsn)* embryos at 2 hpi. The relative quantity of *tnfα*, *il-8* and *il-1b* was examined by qRT-PCR, and expression levels were adjusted for trauma (PBS-solution injection). (Mean±s.e.m., *n* = 30 in each group, triplicated). Statistical significance was determined using the two-tailed Student's *t*-test. *p < 0.05. ***p < 0.001. (*g*) The altered expression of inflammatory cytokines could be rescued by *npsn* overexpression. The relative quantity of *tnfα*, *il-8* and *il-1b* was examined by qRT-PCR, and expression levels were adjusted for trauma (PBS-solution injection). (Mean±s.e.m., *n* = 30 in each group, triplicated). Statistical significance was determined using the one-way ANOVA with Bonferroni's multiple comparisons adjustment. *p < 0.05. ***p < 0.001. n.s., not significant.

To assess whether Npsn enhances the clearance of *E. coli*, we cloned the Npsn*-*coding sequence fused with a 6×Myc tag and driven by the *hsp* promoter into the Tol2 vector (referred to as *pTol2-hsp-Myc-npsn*) (figure 6*a*). This construct was injected into one-cell-stage WT embryos, and the progenies of F0 founders were identified by anti-Myc staining. The F1 Myc^+^ embryos were selected and denoted as the *Tg(hsp:Myc-npsn)* line (figure 6*b*). *Tg(hsp:Myc-npsn)* and WT embryos were subsequently infected with *E. coli* and exposed to heat-shock conditions, followed by recording of survival rates (figure 6*c*). Our findings showed that the *Tg(hsp:Myc-npsn)* line exhibited a significantly higher survival rate relative to the WT variants (figure 6*d*), suggesting that *npsn* overexpression enhanced host immune response to *E. coli* infection in zebrafish embryos.

When we measured kinetic curves associated with *in vivo* bacterial growth of infected embryos, we found that the bacterial burden in *Tg(hsp:Myc-npsn)* embryos was significantly lower than in WT embryos at 1 and 2 dpi (figure 6*e*). The data revealed that *E. coli* proliferated more slowly in the *npsn* overexpression embryos, indicating that *npsn* overexpression could enhance host defence against *E. coli* infection in zebrafish embryos. The expression of inflammatory factors (*tnfα*, *il-8* and *il-1b*) was lower in *Tg(hsp:Myc-npsn)* embryos than in WT controls (figure 6*f*), suggesting the reduced inflammation in *npsn* overexpression embryos. Moreover, overexpressing *npsn* could rescue the altered inflammatory response in *npsn^smu5^* mutant embryos (figure 6*g*), indicating the importance of Npsn in host defence against bacteria.

**Figure 7.**
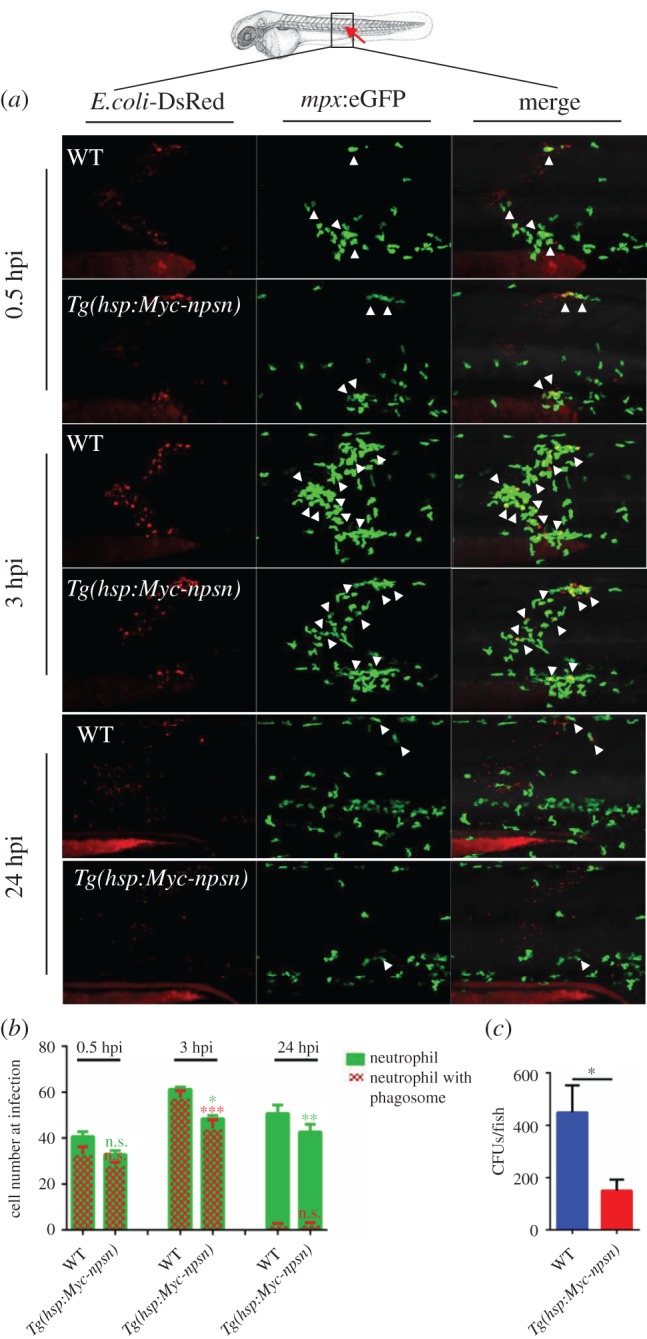
Less neutrophils are recruited at the infection in *Tg(hsp:Myc-npsn)* embryos. (*a*) The infection site of the zebrafish muscle (the red arrow). DsRed^+^ *E. coli* were injected subcutaneously over one somite into WT and *Tg(hsp:Myc-npsn)* embryos with *Tg(mpx:eGFP)* background, and images were captured at 0.5, 3 and 24 hpi. All images are maximum-intensity projection at an interval of 2 μm. White triangles indicated neutrophils with phagosomes. (*b*) Quantification of recruited *mpx:eGFP^+^* neutrophil numbers (green bar) and phagocytosing neutrophil numbers (red net bar) in the infection site at each time point in bacterial injected WT and *Tg(hsp:Myc-npsn)* embryos. Average numbers with means in WT and *Tg(hsp:Myc-npsn)* groups at 0.5, 3, 24 hpi (green bar: 41.0 ± 2.0 versus 32.8 ± 1.5; 61.2 ± 3.1 versus 48.4 ± 3.0; 47.8 ± 4.5 versus 33.0 ± 3.7); (red net bar: 33.0 ± 3.3 versus 28.2 ± 81.3; 57.6 ± 3.0 versus 44.3 ± 3.7; 2.2 ± 0.6 versus 2.4 ± 0.7). (Mean±s.e.m., *n* ≥ 6 in each group, triplicated). Statistical significance was determined using the two-way ANOVA with Bonferroni's multiple comparisons adjustment. *p < 0.05. **p < 0.01. ***p < 0.001. n.s., not significant. (*c*) Bacterial burden of embryos at 24 hpi in *Tg(hsp:Myc-npsn)* embryos and WT controls. (Mean±s.e.m., *n* = 50 in each group, triplicated). Statistical significance was determined using the unpaired *t*-test. *p < 0.05.

In muscle infection assay, neutrophil recruitment was observed and numbers were calculated at 0.5, 3 and 24 hpi. Compared with WT control embryos, *Tg(hsp:Myc-npsn)* embryos had decreased number of recruited neutrophils, as well as decreased phagocytosing neutrophils in the infected site (figure 7*a*,*b*). These results indicated that *npsn* overexpression embryos required less neutrophils to be recruited in clearance of bacteria, supporting that *npsn* overexpression embryos had reduced inflammation during infection and enhanced host immune response to *E. coli* infection. Consistently, bacterial burden in *Tg(hsp:Myc-npsn)* embryos was significantly lower than in WT embryos at 24 hpi (figure 7*c*), which further indicates *npsn* overexpression improved neutrophil-specific antibacterial response in zebrafish embryos.

## 3. Discussion

In this study, we generated an *npsn*-deficient zebrafish mutant and established the *Tg(npsn:EGFP)smu7* transgenic line, which provided a useful tool for studying neutrophil development and behaviour during host wound healing or defence against infection *in vivo*. The CRISPR/Cas9-generated *npsn*-deficient mutants exhibited unaltered neutrophil number and no obvious developmental defects in their neutrophils. When challenged with *E. coli*, *npsn^smu5^* embryos exhibited a lower survival rate and higher bacterial burden, as well as an augmented inflammatory response, relative to WT variants. Additionally, *npsn*-overexpressing zebrafish exhibited higher survival rates, reduced bacterial burden, as well as reduced inflammatory response, when compared with WT variants, indicating that zebrafish Npsn promoted host immune defence against bacterial infection.

Given that Npsn is a component of neutrophil granzymes, *npsn* deficiency affected neutrophil function rather than overall neutrophil development and concentration. *E. coli* is a Gram-negative and non-pathogenic bacterium; however, infection of the zebrafish yolk sac with *E. coli* [28] was lethal at doses as low as 10 CFUs. Additionally, infection of *npsn^smu5^* mutants and their WT controls revealed that *npsn^smu5^* mutants exhibited a lower survival rate and higher bacterial burden when compared with WT, suggesting that *npsn* deficiency impaired host defence against *E. coli* infection*.* Furthermore, during the early stage of infection, the expression of inflammatory factors (*il-1b* [31], *il-8* [30] and *tnfα* [29]) increased significantly in the *npsn^smu5^* embryos. Elevated levels of cytokine transcription might be explained by functional defects associated with neutrophils, resulting in their inability to engulf and degrade invading microbes, thereby leading to greater bacterial burden in the mutant embryos.

Although we confirmed that *npsn* deficiency impaired host defence against bacterial infection, the mechanisms associated with this deficiency are unclear. Npsn is a zinc metalloendopeptidase, and a previous *in vitro* study showed that Npsn hydrolyzes gelatin and fibronectin, which are important components of the extracellular matrix [12,32]. Besides bony fish, Npsn homologues could be found in *Caenorhabditis elegans*, *Drosophila melanogaster* as well as in parasites [33], while no direct mammalian homologues were found (data not shown). By BLAST comparison with the human and mice protein databases, zebrafish Npsn shared about 40% similarity with human and mouse astacin-like metalloendopeptidase, meprin proteinases, bone morphogenetic protein 1 (Bmp1) and tolloid-like protein 1/2, owing to a conserved astacin domain (https://blast.ncbi.nlm.nih.gov/Blast.cgi). These hit proteins exhibited diverse biological functions, including matrix assembly, digestion and signalling pathway activation in developmental morphogenesis and tissue differentiation [34–36]. Here, we found zebrafish Npsn also plays important roles in innate immunity. We found that *npsn-*deficient neutrophils had not lost the ability to engulf the microbe, as neutrophils with phagosomes were observed. And the myeloperoxidase activity was also normal in *npsn-*deficient neutrophils (data not shown). However, embryos with *npsn* deficiency had impaired host defence against bacterial infection, with more neutrophils recruited to the infected site and increased host inflammatory response. Thus it is likely that Npsn may function as a hydrolase to conduct neutrophils for the degradation of engulfed microbes by hydrolysis, or may function as its mammalian counterparts (e.g. Bmps etc.) to play roles as a signal molecule for inflammatory response, which needs further investigation.

In conclusion, this study confirmed that zebrafish Npsn is important to the host immune response against bacterial infection. Our results suggested that *npsn*-deficient and -overexpressing zebrafish could serve as valuable models for *in vivo* investigation of host innate immune response to bacterial pathogens.

## 4. Material and methods

### 4.1. Zebrafish lines and maintenance

All zebrafish lines were raised and maintained under standard conditions [37]. The lines *Tg(mpx:EGFP)* [38], *Tg(mpeg1:EGFP)* [39] and *Tg(lyz:DsRed)* [40] were previously described. Zebrafish embryos were maintained in ‘egg water’ containing 0.002% methylene blue to prevent fungal growth, which was replaced with fresh egg water containing 0.003% *N*-phenylthiourea (Sigma-Aldrich, St Louis, MO, USA) at 10 hpf to 24 hpf to prevent pigmentation.

### 4.2. *npsn*-knockout by CRISPR/Cas9

*npsn*-deletion transcripts were generated using CRISPR/Cas9 technology targeting the fifth exon of the *npsn* gene. The Cas9-targeting sequence was as follows: 5′-GGAGACAT CGCCTTTCCCAG-3′. Cas9 mRNA and genomic RNA were synthesized using the mMESSAGE mMACHINE mRNA transcription-synthesis kit (AM1344; Ambion; Thermo Fisher Scientific, Waltham, MA, USA) using PCR products with the following primer combinations: forward, 5′-TAATACGACTCACTATAGGAGACATCGCCTTTCCCAGGTTTTAGAGCTAGAAATAGC-3′; and reverse, 5′-AGCACCGACTCGGTGCCACT-3′ [41]. Cas9 mRNA (300 ng µl^−1^) and gRNA (guide RNA, 100 ng µl^−1^) were co-injected into one-cell-stage WT embryos. To determine mutation efficiency, genomic DNA was extracted from 24 embryos (three embryos per group), followed by T7 endonuclease I digestion (M0302S; NEB, Ipswich, MA, USA) and Sanger sequencing. The target region was amplified using a forward primer 5′-GGACAGTGCTATTGCGTTTGG-3′ and reverse primer 5′-GCCTTGTTCAATCACTGCTACTTC-3′. The remainder of the embryos were raised to sexual maturity, and positive F0 adults were mated with WT to obtain the F1 generation. Positive F1 adults were detected as the F0 generation, and positive F1 zebrafish with identical mutations were intercrossed to obtain F2 homozygous-mutant and WT offspring. In this programme, *npsn^smu5^* mutants were used for experiments and WT siblings as controls. The *npsn* exon6-Cas9 was designed and synthesized according to a similar protocol to obtain a mutant (−0, +1) (*npsn^smu6^*) harbouring a frameshift mutation.

### 4.3. *In vitro* synthesis of antisense RNA probes and WISH

The antisense RNA probes for *mpx*, *lyz* and *npsn* were prepared by *in vitro* transcription according to a standard protocol [42]. WISH was performed at a hybridization temperature of 65°C as described previously [43].

### 4.4. RNA extraction and qRT-PCR

RNA was isolated using TRIzol reagent (Life Technologies, Carlsbad, CA, USA) according to the manufacturer's instructions. A total of 2 μg RNA was used in a RT reaction using Moloney murine leukaemia virus reverse transcriptase (M1701; Promega, Madison, WI, USA) and oligo-dT (18) according to the manufacturer's instructions. The resulting cDNA was diluted three times for use in qRT-PCR assays. Each 10 μl reaction mixture contained 1 µl cDNA, 200 nM of each gene-specific primer (electronic supplementary material, table S1), and 5 µl of SYBR Green PCR Master Mix (Applied Biosystems, Foster City, CA, USA). Real-time PCR was performed on a LightCycler 96 system (Roche, Basel, Switzerland), and quantitation was performed in triplicate wells. All reactions were normalized against *β-actin*, and melting-curve analysis confirmed the presence of only one PCR product. Significance was determined by using a Student's *t*-test with a significance threshold of *p* < 0.05.

### 4.5. Double fluorescence immunohistochemistry staining

Immunohistochemistry was performed as described previously [44]. To examine the co-staining of GFP or DsRed with *npsn* WISH, embryos were first incubated with an *npsn* antisense probe as described previously, except that the signal was expanded using a TSA Plus fluorescein evaluation kit (NEL741E001KT; PerkinElmer, Waltham, MA, USA) or TSA Plus cyanine 3 evaluation kit (NEL744E001KT; PerkinElmer). For antibody staining, embryos were first stained with goat anti-GFP or rabbit anti-DsRed antibody (1 : 200) at 4°C overnight and subsequently visualized by Alexa Fluor 488 donkey anti-goat (1 : 400) at 4°C overnight or Alexa Fluor 555 donkey anti-rabbit (1 : 400) 4°C overnight, respectively.

### 4.6. Bacterial infection

The DsRed-labelled XL10 *E. coli* cells [33] were expanded at 37°C in a shaker until reaching an optical density of between 1 and 1.5. Bacteria were harvested by centrifugation at 5000*g* for 5 min and resuspended in sterile PBS. The working concentration of *E. coli* was 2 × 10^8^ ml^−1^, and an approximately 0.5 nl bacterial suspension was injected into 2 dpf embryos with 0.02% tricaine. *E. coli* cells (100 CFU) were injected into the yolk sac with a gas manipulator (Havard Apparatus, Holliston, MA, USA) for survival-rate counts. Muscle infection was performed as described previously [22]; *E. coli* cells (10^3^ CFU) were injected subcutaneously over a somite in 2 dpf embryos.

To get a comparable genetic background between the controls and *npsn^smu5^* mutants, the off-spring (F1) from *npsn^smu5/+^* heterozygote (F0) inter-crossing were raised and genotyped for WT pool and mutant pool; the intercrossed off-spring (F2) from each pool were utilized for the infection as WT controls and *npsn^smu5^* mutants, respectively.

### 4.7. Establishment of stable transgenic lines

To establish the *Tg(npsn:EGFP)smu7* line, we cloned the *npsn* regulatory sequence containing putative *npsn* promoter elements by PCR using *npsn*-specific primers (forward, 5′-CCGCTCGAGCAAGCCAAGCAAGAGTTTTACAAG-3′; reverse, 5′-CCCAAGCTTACCATCAATCAGCCATAATTCAGC-3′). The 2-kb DNA sequence upstream of the *npsn* translation start site was identified, placed upstream of GFP, and subcloned into the pTol vector with minimal Tol2 elements and an SV40 polyA sequence to form the *pTol2-npsn-EGFP* construct. To generate the transgenic line, 75 pg of the *pTol2-npsn-EGFP* construct and 25 pg of transposase mRNA were co-injected into zebrafish embryos at the one-cell stage. Founders were selected by fluorescence microscopy and identified by PCR of the transgenic line. Stable F1 *Tg(npsn:EGFP)smu7* embryos were obtained by mating founder fish with AB fish and confirmed by anti-GFP immunostaining. The transgenic line *Tg(hsp:Myc-npsn)* was founded using a similar protocol.

### 4.8. Statistical methods

Calculated data were recorded and analysed using GraphPad Prism 6 software (GraphPad Software, La Jolla, CA, USA). Student's *t*-test was used for comparisons between two groups, and one-way or two-way analysis of variance was used for comparisons among multiple groups, whereas comparison of survival curves was performed using the log-rank test. Differences were considered significant at *p* < 0.05. Data are expressed as mean ± s.e.m.

## Supplementary Material

Supplementary Materials

## Data accessibility

The datasets supporting this article have been uploaded as part of the electronic supplementary material.
