# Supplementary material for: Zebrafish nephrosin helps host defence against Escherichia coli infection
Source: Open Biol. 2017 Aug 23;7(8):170040. doi: 10.1098/rsob.170040 (PMC5577445; doi:10.1098/rsob.170040)
Supplement: Supplementary Materials [file rsob170040supp1.pdf]

**Supplementary Materials**

**Figure S1. Generation and characterization of the *Tg(npsn:EGFP)smu7* line.**

A. Construction of the *Tg(npsn:EGFP)smu7* transgenic plasmid. A 2-kb upstream sequence of the zebrafish *npsn* gene (pink bar; referred to as the *npsn* promoter) was cloned and inserted prior to the GFP gene (green bar) and flanked by the two TP sites (yellow bar) in the Tol2 transgenic plasmid. B. The expression pattern of GFP signals in *Tg(npsn:EGFP)smu7* at 3 dpf. C. Double fluorescent staining for GFP and *npsn* WISH in *Tg(npsn:EGFP)smu7* at 3 dpf. (D–F) and (D'–F'). Inter-crossing of *Tg(npsn:EGFP)smu7* and *Tg(lyz:DsRed)* lines to reveal overlapping populations. $npsn^+$ cells overlapped 100% in the thymus (D and D'), 87.09 ± 9.45% in the AGM (E and E'), and 90.05 ± 2.85% in the PBI (F and F') with $mpx^+$ cells. White arrows indicate non-overlapping signals. G. The *npsn* promoter drives expression specifically in neutrophils. $GFP^+$ cells sorted from *Tg(npsn:EGFP)smu7* and *Tg(mpx:EGFP)*. qRT-PCR showed neutrophil markers (*lyz*, *mpx*, and *npsn*) expressed at higher levels in $npsn^+$ cells as compared with levels observed in $mpx^+$ cells. The # represents "undetected", [Mean ± SEM, $n \geq 200$ in each group, triplicated], and statistical significance was determined using the two-tailed Student's $t$ test. ***$p < 0.001$.

**Figure S2. Decreased *npsn* mRNA levels in CRISPER/Cas9-mediated *npsn*-knockout lines.**

A. The other Cas9 target was chosen on *npsn* exon6 to obtain a (−0, +1) mutant ($npsn^{smu6}$) containing a frameshift mutation. B. *npsn* WISH results showed

significantly decreased *npsn* mRNA in $npsn^{smu5}$ mutant embryos at 24 hpf, 36 hpf, and 2 dpf. C. *npsn* WISH results showed *npsn* expression was also significantly decreased in $npsn^{smu6}$ embryos.

**Figure S3. The expression of *npsn* is unaffected by the infection.**

Relative expression of *npsn* in sorted neutrophils of *E.coli*-infected and PBS-injected *Tg(mpx:EGFP)* embryos at 2 hpi. [Mean ± SEM, $n \geq 200$ in each group, triplicated]. Statistical significance was determined using the two-tailed Student's *t* test. ns, not significant.

**Table S1. Primers used in this study**

| Gene name | primer |
|---|---|
| *b-actin* | F 5'-AAGATCAAGATCATTGCTCCC-3' |
| | R 5'-GAGAGGTTTAGGTTGGTCGT-3' |
| *mpx* | F 5'-CCGAGATGGCGATAGGTTG-3' |
| | R 5'-TCGAGATCAAAAGCTGGGATA-3' |
| *lyz* | F 5'-AAAGCAGGTTTAAGACCCAC-3' |
| | R 5'-CCAGGTTTCCCATGATTTCAG-3' |
| *mfap4* | F 5'-GTTTACACCATCTATCCAGCC-3' |
| | R 5'-GTTCTCTAGTCCCAGCCA-3' |
| *mpeg1* | F 5'-GGGTTCAAGTCCGTAACCA-3' |
| | R 5'-CAACACTTGTGATGACATGGG-3' |
| *il-1b* | F 5'-GTACTCAAGGAGATCAGCGG-3' |
| | R 5'-CTCGGTGTCTTTCCTGTCCA-3' |
| *il-8* | F 5'-TGTTTTCCTGGCATTTCTGACC-3' |
| | R 5'-TTTACAGTGTGGGCTTGGAGGG-3' |

| | |
|---|---|
| *tnfa* | F 5'-GCTGGATCTTCAAAGTCGGGTGTA-3' |
| | R 5'-TGTGAGTCTCAGCACACTTCCATC-3' |
| *tnfb* | F 5'-TCACTTGCATGGTGACCCTTC-3' |
| | R 5'-GACCATCCTTAGGAATGATGATCTCG-3' |
| *npsn-front* | F 5'-AGCTGTCATCATGTACCTGTTGG-3' |
| | R 5'-CTTTGTGAAGTTACCACCAGTCTTC-3' |
| *npsn-middle* | F 5'-CAAACATGCAGGAGAGAACACAG-3' |
| | R 5'-CATCTCTGCTTCTGTCCCATTTG-3' |
| *npsn-later* | F 5'-GTCTGAATCTGGCTGCTATTCATATTTAG-3' |
| | R 5'-ATGAAGGAGCTCGTGCTGG-3' |

Fig. S1

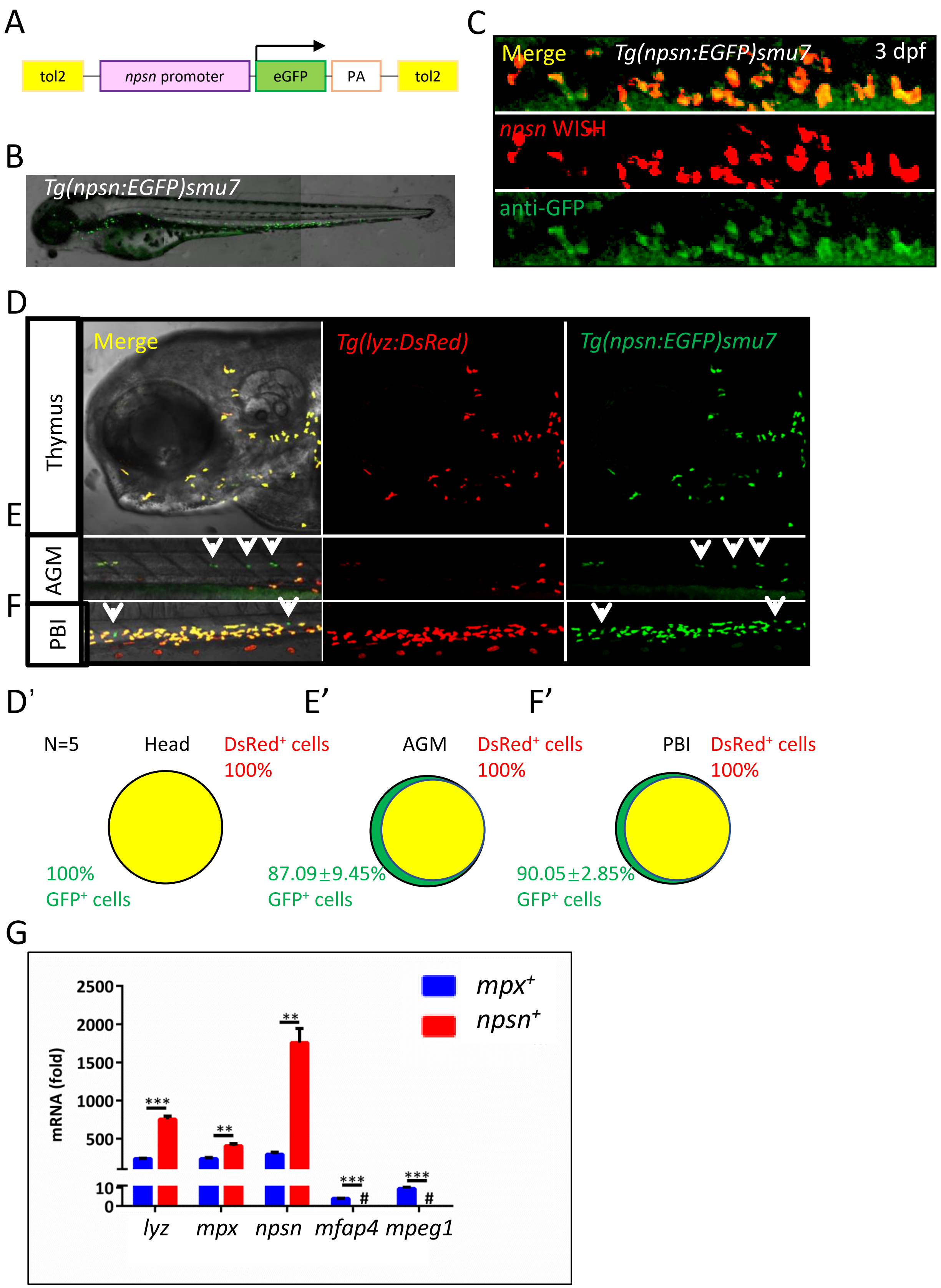
A
tol2
npsn promoter
eGFP
PA
tol2
B
Tg(npsn:EGFP)smu7
C
Merge
Tg(npsn:EGFP)smu7
3 dpf
npsn WISH
anti-GFP
D
E
F
Thymus
AGM
PBI
Merge
Tg(lyz:DsRed)
Tg(npsn:EGFP)smu7
D'
E'
F'
N=5
Head
DsRed+ cells
100%
100%
GFP+ cells
AGM
DsRed+ cells
100%
87.09±9.45%
GFP+ cells
PBI
DsRed+ cells
100%
90.05±2.85%
GFP+ cells
G
mpx+
npsn+
mRNA (fold)
2500
2000
1500
1000
500
10
0
***
**
**
***
***
#
#
lyz
mpx
npsn
mfap4
mpeg1

Fig. S2

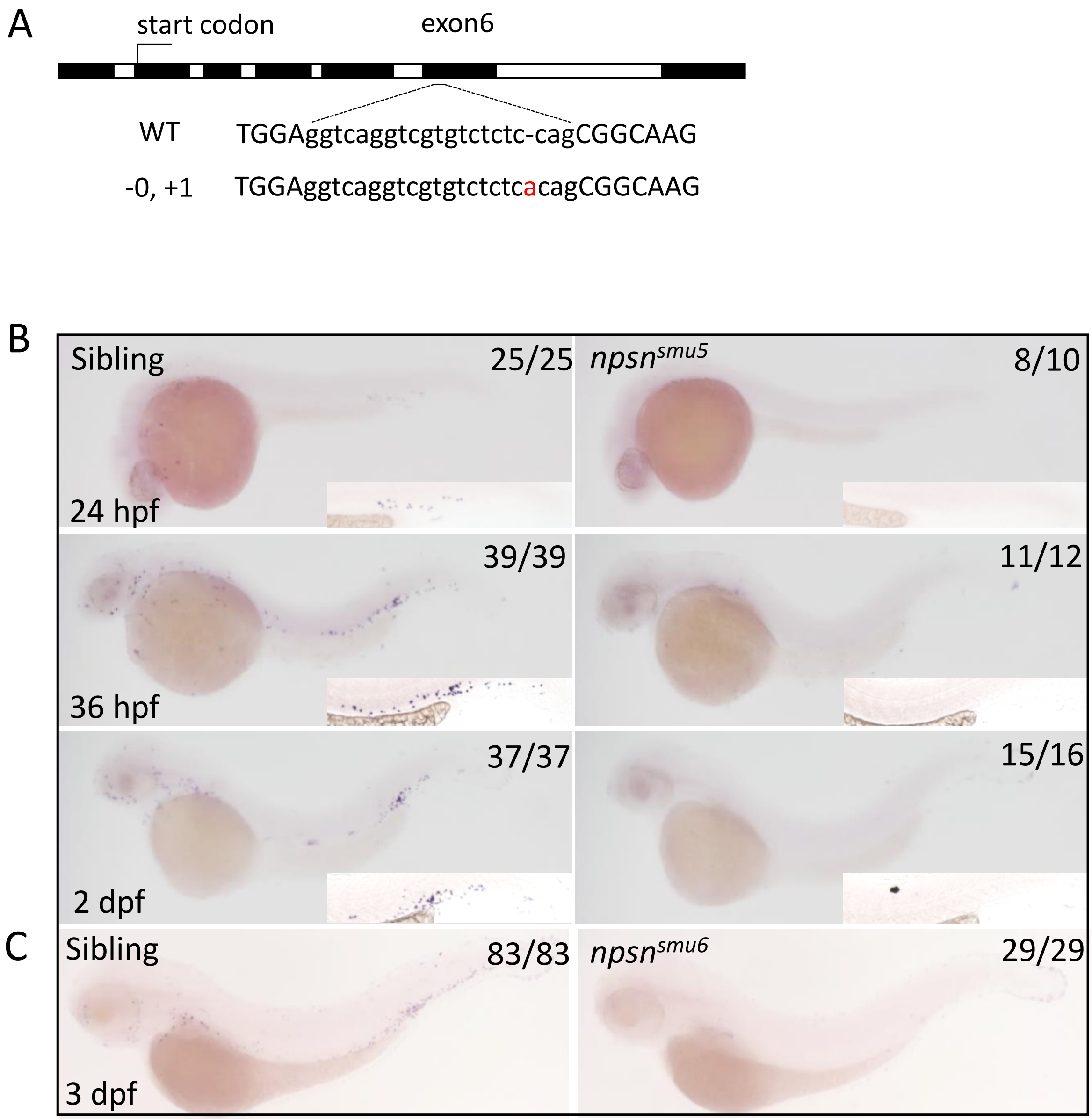
A
start codon
exon6
WT
TGGAggtcaggtcgtgtctctc-cagCGGCAAG
-0, +1
TGGAggtcaggtcgtgtctctcacagCGGCAAG
B
Sibling
25/25
npsn^smu5
8/10
24 hpf
39/39
11/12
36 hpf
37/37
15/16
2 dpf
C
Sibling
83/83
npsn^smu6
29/29
3 dpf

Fig. S3

A

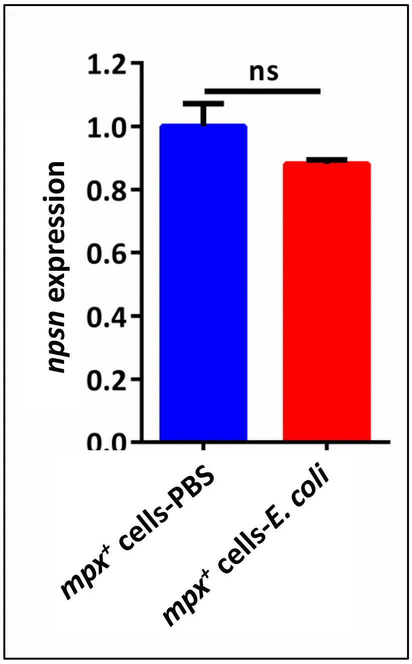
ns
1.2
1.0
0.8
0.6
0.4
0.2
0.0
npsn expression
mpx+ cells-PBS
mpx+ cells-E. coli
